# Swallowing rehabilitation in aspiration pneumonia: A scoping review of compensatory strategies and exercise training effectiveness

**DOI:** 10.1002/jgf2.70047

**Published:** 2025-07-15

**Authors:** Akihito Ueda, Kanji Nohara

**Affiliations:** ^1^ Medical Corporation Toujinkai Fujitate Hospital Osaka Japan; ^2^ Doctoral Program in Pharmaceutical Sciences, Graduate School of Pharmaceutical Sciences Teikyo Heisei University Tokyo Japan; ^3^ Department of Rehabilitation for Orofacial Disorders Osaka University Graduate School of Dentistry Osaka Japan

**Keywords:** aspiration pneumonia, compensatory strategies, exercise training, oropharyngeal dysphagia, swallowing rehabilitation

## Abstract

Aspiration pneumonia (AP) is a major concern in aging populations. AP management requires a comprehensive, multimodal approach—notably including swallowing rehabilitation, oral care, and risk factor management. This scoping review evaluates evidence on swallowing rehabilitation for AP, which consists of compensatory strategies and exercise training. A systematic literature search identified 23 eligible studies from 522 articles; four directly investigated swallowing rehabilitation in AP, while 19 focused on patients with dysphagia due to stroke, neurological diseases, or aging. Two large‐scale retrospective AP‐specific studies demonstrated a correlation between rehabilitation and improved oral intake but did not evaluate AP prevention or specify effective components. In one study, water jelly ingestion reduced AP incidence. Another study demonstrated the benefits of texture‐modified diets in reducing aspiration risk, although AP prevention was not assessed. While clinically compensatory strategies remain foundational, current evidence does not support routine exercise‐based interventions for AP prevention. Further high‐quality studies are needed.

## 1 INTRODUCTION

Aspiration pneumonia (AP) represents a significant health challenge in aging societies, particularly in Japan, which faces a more advanced stage of population aging than other countries and experiences disproportionately high hospitalization rates compared to Western countries. According to the British Thoracic Society (BTS) Clinical Statement on Aspiration Pneumonia,^1^ AP accounts for more than 60% of community‐acquired pneumonia hospitalizations in Japan, compared to only 5%–15% in Western countries. In response to this burden, the Japanese Respiratory Society (JRS) revised its guidelines,^2^ placing substantial emphasis on AP management and care.

Komiya et al.^3^ revealed in their single‐center study that approximately 73% of older patients with pneumonia were treated by non‐pulmonologists, including general internists and primary care physicians. Their research showed no significant differences in 30‐day or 90‐day mortality between patients treated by pulmonologists and those treated by non‐pulmonologists, suggesting that with appropriate guidance, primary care physicians can effectively manage pneumonia in older populations. Since pneumonia in older patients is mostly considered to be AP, these findings highlight the important role that primary care physicians play in the frontline management of AP in Japan.

The JRS guidelines emphasize the importance of swallowing rehabilitation for patients with AP, as reported by Momosaki.^4^ The review, which builds on his original article,^5^ outlines the fundamental components of swallowing rehabilitation, including behavioral and dietary modifications, as well as direct and indirect swallowing exercises:

- Behavioral modification: including positioning for feeding.
- Dietary modification: including texture modification and thickened liquids.
- Direct swallowing exercises: including intentional swallowing and supraglottic swallowing technique.
- Indirect swallowing exercises: including head‐lift exercises and range‐of‐motion exercises for the neck.

The concept of ‘swallowing rehabilitation’ itself varies in literature. The BTS Clinical Statement considers the above behavioral modification and dietary modification as compensatory strategies rather than rehabilitation. Similarly, the European Society for Swallowing Disorders–European Union Geriatric Medicine Society (ESSD‐EUGMS) white paper distinguishes between compensatory strategies (aimed at avoiding aspiration through modifications of food and posture) and rehabilitative approaches (aimed at improving swallowing function).^6^

In this paper, for clarity and to avoid terminological ambiguity, we refer to the ESSD‐EUGMS white paper terms “rehabilitative approaches” as “exercise training”. We define swallowing rehabilitation as consisting of both exercise training and compensatory strategies. This exercise training corresponds to what Momosaki et al.^4^, ^5^ described as direct swallowing exercises (including intentional swallowing and supraglottic swallowing technique) and indirect swallowing exercises (including head‐lift exercises and range‐of‐motion exercises for the neck).

Despite being widely implemented in AP management, swallowing rehabilitation's conceptual framework remains inconsistent, leading to variability in clinical application. This ambiguity has resulted in clinical situations where exercise training is applied without clear evidence of efficacy for the prevention of AP, sometimes imposing unnecessary burdens on healthcare providers and patients.

To develop evidence‐based recommendations, it is essential to establish whether existing studies support compensatory strategies, exercise training, or both. Additionally, it remains unclear whether swallowing rehabilitation for the prevention of AP has been rigorously investigated in AP‐specific populations or primarily studied in related conditions such as stroke, neurological diseases, or general geriatric cohorts. For instance, the BTS Clinical Statement highlights Thermal‐Tactile Stimulation as a potentially effective intervention, yet supporting studies^7^ predominantly focus on patients with Parkinson's disease, raising concerns about the generalizability of these findings to patients with AP.

To address these uncertainties, we conducted a scoping review to systematically map existing research, identifying conceptual inconsistencies and knowledge gaps. This review further examines swallowing rehabilitation interventions for AP, classifying them as compensatory or exercise‐based and evaluating their impacts on clinical outcomes.

## 2 MATERIALS AND METHODS

### 2.1 Study design

This study was conducted as a scoping review, a methodological approach designed to systematically map key concepts, identify research trends, and highlight knowledge gaps within a given field.^8^ This approach was selected to provide a comprehensive evaluation of the literature on swallowing rehabilitation in AP and assess the existing evidence's variability, validity, and limitations.

This scoping review was conducted in accordance with the Preferred Reporting Items for Systematic Reviews and Meta‐Analyses extension for Scoping Reviews (PRISMA‐ScR) Checklist (see Material S1).

### 2.2 Search strategy

A comprehensive literature search was conducted using MEDLINE and the Cochrane Library, with the search performed on March 12, 2025. The search included all studies published up to December 31, 2024. The search strategy was deliberately broad to maximize the inclusion of relevant studies. In MEDLINE, the search used the query (“aspiration pneumonia”) AND (“rehabilitation” OR “swallowing therapy” OR “swallowing training” OR “dysphagia therapy” OR “dysphagia training”) NOT “case reports”[Publication Type]. Notably, we used the general term “rehabilitation” rather than limiting it to specific terms like “swallowing rehabilitation” or “dysphagia rehabilitation” to ensure maximum inclusion of potentially relevant articles. This approach likely enabled us to capture studies using various terminologies for swallowing interventions, including both swallowing and dysphagia‐focused rehabilitation approaches. Additional filters excluded preprints, restricted results to English‐language studies conducted on humans, and ensured relevance to clinical rehabilitation. The Cochrane Library was searched using the same core terms, without additional exclusions.

In total, the search retrieved 522 references, including 521 from MEDLINE and 1 from the Cochrane Library.

### 2.3 Study selection

Studies were selected based on the Patient, Concept, and Context (PCC) criteria, ensuring alignment with the research focus. The patient criterion included patients diagnosed with AP as well as those with a high risk of AP due to underlying swallowing difficulties, such as post‐stroke patients, those with neurodegenerative diseases, and older patients with dysphagia. This broader inclusion was justified for three main reasons: (1) These populations share similar pathophysiological mechanisms of dysphagia with AP patients; (2) the limited number of studies specifically focusing on AP necessitated broadening our scope to include these related conditions; and (3) interventions used in these settings are often directly applicable to AP prevention due to the common underlying mechanisms of dysphagia.

**FIGURE 1:**
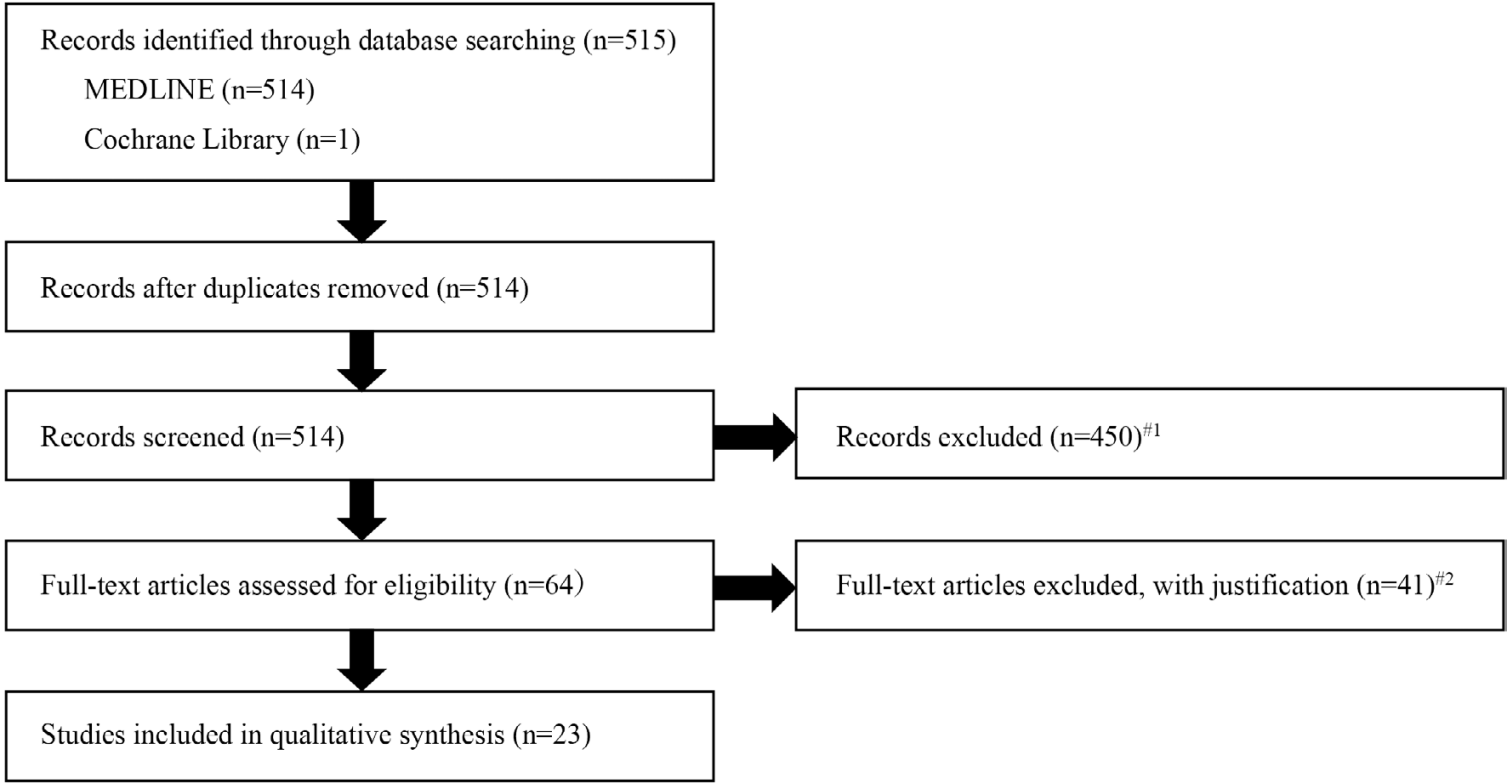
PRISMA Flow Diagram of Study Selection.

The title and abstract screening was initially conducted by one researcher and subsequently verified by a second researcher. Articles meeting the inclusion criteria underwent full‐text review, and two researchers resolved ambiguous cases by consensus. The study selection processes adheres to the PRISMA framework, as illustrated in Figure 1.

A total of 522 articles were initially identified. Following screening, 456 articles were excluded (#1) for the following reasons:

- 15 articles due to publication type (9 comments, 3 letters, 2 case reports, and 1 editorial).
- 19 pediatric studies.
- Remaining records focused on postoperative dysphagia or did not address rehabilitation effectiveness.

The remaining 66 articles underwent full‐text evaluation. From the full‐text evaluation, 43 articles were excluded (#2) for the following reasons:

- 23 review articles, systematic reviews, meta‐analyses, and policy forum papers.
- 5 articles focused on physical rehabilitation.
- 2 articles focused on interventions by multidisciplinary swallowing teams.
- 4 articles that did not address rehabilitation effectiveness.
- 1 article on individuals without dysphagia (healthy individuals).
- 4 protocol papers without results.
- 4 case series.

After the full‐text review, 23 studies were retained for the final analysis.

### 2.4 Terminology

To ensure consistency throughout the manuscript, we applied the following operational definitions for key terms:

- Swallowing rehabilitation: The overarching term encompassing both compensatory strategies and exercise‐based interventions aimed at managing dysphagia and preventing aspiration.
- Compensatory strategies: Techniques that work around swallowing impairments without specifically targeting physiological improvement, including postural adjustments (e.g., chin‐down posture), food texture modifications, and thickened liquids.
- Exercise training: The category of interventions specifically designed to improve swallowing physiology and function through active exercises and training protocols. This is our operational term for what the ESSD–EUGMS white paper terms “rehabilitative approaches.”
- Swallowing exercises: Specific exercise techniques and protocols within the exercise training category, such as the Shaker exercise, head‐lift exercise, or supraglottic swallowing technique. These are concrete implementations of exercise training principles.

This operational classification allows us to analyze the evidence while acknowledging the terminological variations in the literature, including the broader use of “swallowing rehabilitation” in Japanese clinical context as described by Momosaki et al.^4^, ^5^

These definitions were applied consistently throughout our analysis to categorize and evaluate the interventions described in the included studies.

### 2.5 Data collection and analysis

Data was extracted from each study, capturing study design, target population, intervention characteristics, and measured outcomes. The extracted findings were analyzed to identify trends in intervention approaches, classify studies based on target populations, and assess whether interventions were categorized as compensatory or exercise‐based strategies. We acknowledge that some interventions, particularly in the supplementary studies, were not strictly traditional swallowing rehabilitation techniques, but these studies were included as the techniques targeted functions related to swallowing safety and effectiveness. The consistency of data extraction was ensured through independent review and verification by a second researcher.

## 3 RESULTS

The database search identified 522 articles, of which 23 met the inclusion criteria after screening (Figure 1). These studies were categorized into 4 primary studies directly investigating swallowing rehabilitation in patients with AP (Table 1, studies A1–A4) and 19 supplementary studies examining dysphagia rehabilitation in other populations, with potential implications for AP management (Table 2). The supplementary studies were further stratified into three subgroups based on the studied population: post‐stroke patients (B1–B13), patients with neurological or neuromuscular diseases (C1–C3), and older patients with dysphagia (D1–D3).

**TABLE 1:** Primary studies on swallowing rehabilitation for aspiration pneumonia.

No.	Authors (year)	Title	Journal, volume (number): Pages	Study design	Key findings
A‐1	Otaka Y, et al. (2024)	Early swallowing rehabilitation and promotion of total oral intake in patients with aspiration pneumonia: A retrospective study	*PLoS One*, 19(1):e0296828	Retrospective cohort study	Early rehabilitation reduced mortality and improved oral intake at discharge
A‐2	Morita A, et al. (2022)	Effectiveness of Water Jelly Ingestion for Both Rehabilitation and Prevention of Aspiration Pneumonia in Elderly Patients With Moderate to Severe Dysphagia	*J Clin Gastroenterol*, 56(2):e109‐e113	Randomized controlled trial	Water jelly ingestion reduced AP incidence compared to control group
A‐3	Shimizu A, et al. (2020)	Impact of Multiple Texture‐Modified Diets on Oral Intake and Nutritional Status in Older Patients with Pneumonia: A Retrospective Cohort Study	*Dysphagia*, 35(4):574–582	Retrospective cohort study	Multiple texture‐modified diets improved swallowing function and nutritional status
A‐4	Momosaki R, et al. (2015)	Effect of dysphagia rehabilitation on oral intake in elderly patients with aspiration pneumonia	*Geriatr Gerontol Int*, 15(6):694–699	Retrospective cohort study	Swallowing rehabilitation associated with higher rates of complete oral intake at discharge

**TABLE 2:** Supplementary studies on swallowing rehabilitation in other patient groups.

No.	Authors (year)	Title	Journal, volume (number): Pages	Study design	Key findings
B‐1	Yoo SD, et al. (2023)	Assessing the effect of transcranial magnetic stimulation on peak cough flow in patients with supratentorial cerebral infarction: A retrospective cohort study	*Medicine*, 102(17):e33689	Retrospective cohort study	Transcranial magnetic stimulation improved peak cough flow in post‐stroke patients
B‐2	Kaylor SA, et al. (2023)	Clinical outcomes associated with speech, language and swallowing difficulties post‐stroke	*S Afr J Commun Disord*, 70(1):e1–e15	Retrospective observational study	Swallowing therapy improved oral intake ability in post‐stroke patients
B‐3	Pelczarska A, et al. (2020)	The cost‐effectiveness of food consistency modification with xanthan gum‐based Nutilis Clear in patients with post‐stroke dysphagia in Poland	*BMC Health Serv Res*, 20(1):552	Modeling study	Xanthan gum‐based food modification reduced aspiration risk in post‐stroke dysphagia
B‐4	Krajczy E, et al. (2019)	Assessment of the effects of dysphagia therapy in patients in the early post‐stroke period: a randomized controlled trial	*Neurol Neurochir Pol*, 53(6):428–434	Randomized controlled trial	15‐day dysphagia therapy improved swallowing reflexes in early post‐stroke patients
B‐5	Tarameshlu M, et al. (2019)	The effect of repetitive transcranial magnetic stimulation combined with traditional dysphagia therapy on poststroke dysphagia: a pilot double‐blinded randomized‐controlled trial	*Int J Rehabil Res*, 42(2):133–138	Randomized controlled trial	Repetitive transcranial magnetic stimulation with traditional therapy improved dysphagia in post‐stroke patients
B‐6	Simonelli M, et al. (2019)	A stimulus for eating. The use of neuromuscular transcutaneous electrical stimulation in patients affected by severe dysphagia after subacute stroke: A pilot randomized controlled trial	*NeuroRehabilitation*. 44(1):103–11	Randomized controlled trial	Neuromuscular electrical stimulation improved oral intake in severe post‐stroke dysphagia.
B‐7	Choi JB, et al. (2017)	Effects of Shaker exercise in stroke survivors with oropharyngeal dysphagia	*NeuroRehabilitation*. 41(4):753–757	Quasi‐experimental study	Shaker exercise reduced aspiration risk in stroke survivors with dysphagia
B‐8	Nakazora T, et al. (2017)	Intervention by Speech Therapists to Promote Oral Intake of Patients with Acute Stroke: A Retrospective Cohort Study	*J Stroke Cerebrovasc Dis*, 26(3):480–487	Retrospective cohort study	Speech therapist intervention promoted oral intake in acute stroke patients
B‐9	Li W, et al. (2017)	Effects of extended in‐patient treatment training on outcome of post‐stroke dysphagia	*Eur Rev Med Pharmacol Sci*, 21(24):5711–5716	Prospective controlled study	Extended in‐patient treatment improved the outcome of post‐stroke dysphagia.
B‐10	Terré R, et al. (2012)	Effectiveness of chin‐down posture to prevent tracheal aspiration in dysphagia secondary to acquired brain injury. A videofluoroscopy study	*Neurogastroenterol Motil*, 24(5):414–419	Prospective observational study	Chin‐down posture prevented tracheal aspiration in acquired brain injury dysphagia
B‐11	Michou E, et al. (2012)	Targeting unlesioned pharyngeal motor cortex improves swallowing in healthy individuals and after dysphagic stroke	*Gastroenterology*, 142(1):29–38	Quasi‐experimental study	Targeting unlesioned pharyngeal motor cortex improved swallowing after dysphagic stroke
B‐12	Selley WG, et al. (1995)	Dysphagia following strokes: clinical observations of swallowing rehabilitation employing palatal training appliances	*Dysphagia*, 10(1):32–35	Prospective observational study	Palatal training appliances improved swallowing rehabilitation following strokes
B‐13	DePippo KL, et al. (1994)	Dysphagia therapy following stroke: a controlled trial	*Neurology*, 44(9):1655–1660	Randomized controlled trial	Comparison of different intensities of dysphagia therapy showed no significant difference in pneumonia incidence
C‐1	Claus I, et al. (2021)	Expiratory Muscle Strength Training for Therapy of Pharyngeal Dysphagia in Parkinson's Disease	*Mov Disord*, 36(8):1815–1824	Randomized controlled trial	Expiratory muscle strength training improved swallowing safety in Parkinson's patients.
C‐2	Solazzo A, et al. (2011)	Search for compensation postures with videofluoromanometric investigation in dysphagic patients affected by amyotrophic lateral sclerosis	*Radiol Med*, 116(7):1083–1094	Prospective observational study	Specific compensatory postures reduced aspiration in ALS patients with dysphagia
C‐3	Troche MS, et al. (2010)	Aspiration and swallowing in Parkinson disease and rehabilitation with EMST: a randomized trial	*Neurology*, 75(21):1912–1919	Randomized controlled trial	Expiratory muscle strength training reduced aspiration in Parkinson's disease patients
D‐1	Kyodo R, et al. (2020)	Pureed diets containing a gelling agent to reduce the risk of aspiration in elderly patients with moderate to severe dysphagia: A randomized, crossover trial	*Medicine*, 99(31):e21165	Randomized crossover trial	Pureed diets with gelling agent reduced aspiration risk in elderly patients with dysphagia
D‐2	Rogus‐Pulia N, et al. (2016)	Effects of Device‐Facilitated Isometric Progressive Resistance Oropharyngeal Therapy on Swallowing and Health‐Related Outcomes in Older Adults with Dysphagia	*J Am Geriatr Soc*, 64(2):417–424	Prospective intervention study	Progressive resistance oropharyngeal therapy improved swallowing function in older adults with dysphagia
D‐3	Ueda K, et al. (2004)	Effects of functional training of dysphagia to prevent pneumonia for patients on tube feeding	*Gerodontology*, 21(2):108–111	Quasi‐experimental study	Swallowing exercises with oral care reduced pneumonia incidence in tube‐fed patients

### 3.1 Studies directly investigating swallowing rehabilitation in AP

Four studies specifically examined swallowing rehabilitation interventions in patients with AP.

Otaka et al.^9^ (A‐1) conducted a retrospective study evaluating the impact of early swallowing assessment and rehabilitation on clinical outcomes. The protocol was associated with reduced in‐hospital mortality and a lower risk of oral intake failure at discharge. However, the study did not specify whether compensatory strategies or exercise training contributed to these outcomes.

Momosaki et al.^5^ (A‐4) performed a large‐scale retrospective study using a national inpatient database to assess the effects of swallowing rehabilitation on oral intake in older patients with AP. Patients who received rehabilitation had significantly higher rates of complete oral intake at discharge than those who did not. Like Otaka et al., this study did not detail specific rehabilitation protocols, limiting its applicability to clinical practice.

Morita et al.^10^ (A‐2) conducted a randomized controlled trial investigating the impact of post‐meal water jelly ingestion on AP incidence. Their findings demonstrated that water jelly ingestion significantly reduced the number of new AP cases. This intervention functions as a compensatory strategy, clearing oral and pharyngeal residue rather than improving the underlying swallowing physiology.

Shimizu et al.^11^ (A‐3) performed a retrospective cohort study evaluating the impact of multiple texture‐modified diets in older patients with pneumonia. Although the study was not exclusively focused on AP, its relevance was inferred due to the high likelihood of AP cases in the study population. Facilities offering a broader range of texture modifications, which correspond to compensatory strategies, demonstrated significantly higher improvement rates in swallowing function and nutritional status than those with fewer modifications.

### 3.2 Swallowing rehabilitation in non‐AP populations: Implications for AP management

While not specifically focused on AP, 19 studies provided relevant insights into swallowing rehabilitation that may inform AP management. These studies investigated interventions in three populations: post‐stroke patients, patients with neurological or neuromuscular diseases, and older patients with dysphagia. These populations were included in our analysis because they have a high risk of AP due to their underlying swallowing difficulties or dysphagia.

Among these studies, the one by Ueda et al.^12^ (D‐3) was the only study to directly evaluate pneumonia prevention, demonstrating that weekly swallowing exercises combined with oral care (interventions focused on oral hygiene and dental care, which are distinct from swallowing rehabilitation) significantly reduced pneumonia incidence in tube‐feeding patients with a small number of participants.

Krajczy et al.^13^ (B‐4) conducted a randomized controlled trial in early post‐stroke patients, using a 15‐day dysphagia therapy program with swallowing reflex stimulation and muscle exercises, showing improvement in swallowing reflexes and oral intake ability, while Choi et al.^14^ (B‐7) demonstrated that Shaker exercise significantly reduced aspiration risk and improved oral intake ability in stroke survivors with oropharyngeal dysphagia, yet neither study specifically assessed pneumonia prevention outcomes.

Additional studies explored compensation strategies, with Terré et al.^15^ (B‐10) demonstrating the effectiveness of chin‐down posture in preventing aspiration for patients with acquired brain injury, and Pelczarska et al.^16^ (B‐3) and Kyodo et al.^17^ (D‐1) showing that modified food consistency and use of gelling agents could significantly reduce aspiration risk. Studies by Nakazora et al.^18^ (B‐8), Li et al.^19^ (B‐9), and Kaylor et al.^20^ (B‐2) reported improvements in oral intake ability and swallowing function through various swallowing rehabilitation interventions in post‐stroke patients. Earlier studies by Selley et al.^21^ (B‐12) examined the use of palatal training appliances in stroke patients, while DePippo et al.^22^ (B‐13) conducted controlled trials comparing different intensities of swallowing rehabilitation interventions. Other studies included Solazzo et al.^23^ (C‐2) who examined compensatory postures for amyotrophic lateral sclerosis (ALS) patients with dysphagia, and Rogus‐Pulia et al.^24^ (D‐2) who investigated isometric progressive resistance oropharyngeal therapy in older patients with dysphagia, showing improvements in swallowing function. Several studies investigated novel therapeutic approaches for dysphagia rehabilitation, including transcranial magnetic stimulation^25^, ^26^, ^27^ (B‐1, B‐5, B‐11), neuromuscular electrical stimulation^28^ (B‐6), and expiratory muscle strength training^29^, ^30^ (C‐1, C‐3). While the studies on compensatory strategies and novel therapeutic approaches reported improvements in swallowing parameters and function, none provided sufficient evidence that these functional improvements translated directly to prevention of AP.

## 4 DISCUSSION

Despite the significant clinical burden of AP, particularly in aging populations, this scoping review identified only four studies directly investigating swallowing rehabilitation in patients with AP. While two retrospective studies reported positive associations between swallowing rehabilitation and clinical outcomes, the absence of detailed rehabilitation protocols makes it unclear whether compensatory strategies or exercise training contributed to these benefits. The study on water jelly ingestion demonstrates the effectiveness of compensatory strategies, with its mechanism focused on clearing oral and pharyngeal residue to reduce aspiration risk rather than improving swallowing function. Similarly, the study on texture‐modified diets also supports compensatory strategies. Collectively, current evidence for AP prevention primarily supports compensatory approaches, while evidence for exercise‐based interventions remains insufficient to support their use in clinical practice.

Most studies focused on specific, well‐defined patient populations rather than the heterogeneous population of patients with AP. Among the 19 supplementary studies (Table 2), 13 examined post‐stroke patients, 3 focused on neurological diseases, and 3 investigated dysphagia rehabilitation in older patients with dysphagia.

A significant limitation of the current evidence base is the relatively small number of studies specifically addressing patients with AP. Much of our understanding of swallowing rehabilitation comes from research on related populations such as post‐stroke patients, those with neurodegenerative diseases, and older individuals with dysphagia. However, substantial caution must be exercised when extrapolating these findings to patients with AP due to fundamental differences in pathophysiology and rehabilitation goals.

The pathophysiology underlying dysphagia varies considerably across these populations. Post‐stroke dysphagia typically results from acute neurological damage with recovery potential, while patients with AP often present with multifactorial dysphagia involving frailty, cognitive impairment, and various comorbidities. Rehabilitation goals also differ substantially—post‐stroke interventions often aim for functional recovery, while AP management may focus more on risk reduction and safety maintenance. These differences significantly limit the generalizability of findings from non‐AP populations. While compensatory strategies may be more universally applicable, exercise‐based interventions targeting neuromotor functions might produce highly variable outcomes depending on the underlying condition. Therefore, clinicians should interpret evidence from related populations with considerable caution and prioritize interventions with demonstrated efficacy specifically in patients with AP.

Compensatory strategies, such as chin‐down posture and texture modifications, may be more generalizable across different patient groups as they address the mechanical aspects of swallowing that are commonly affected regardless of the underlying condition. These interventions focus on modifying the swallowing environment rather than the neurophysiological mechanisms, potentially making them applicable across various dysphagia etiologies.

In contrast, exercise‐based interventions may have more condition‐specific effects, as they target underlying neuromotor function that may be affected differently in various conditions. For example, exercises that prove effective in post‐stroke dysphagia might not yield the same benefits in patients with AP due to frailty or neurodegenerative diseases. The effectiveness of these approaches likely depends on factors such as the patient's cognitive status, overall physical condition, and the specific pathophysiology of their dysphagia.

Future research should aim to validate findings from these related populations specifically in AP patients to determine which interventions are truly generalizable to AP management.

Of these studies, only three specifically focused on exercise‐based approaches: These included a randomized controlled trial on comprehensive dysphagia therapy in early post‐stroke patients,^13^ a study on the Shaker exercise combined with conventional therapy in stroke survivors,^14^ and an investigation of regular swallowing exercises in tube‐fed patients.^12^ Of these studies, only Ueda et al.^12^ specifically evaluated pneumonia prevention, reporting a reduction in pneumonia incidence with weekly swallowing exercises. However, the small sample size of this study limits the generalizability of its findings. The other two studies demonstrated improvements in swallowing function, but their impact on pneumonia prevention was not directly assessed. While these studies demonstrated improvements in swallowing function, their role in reducing AP incidence remains unclear. Current evidence does not support the routine use of exercise‐based interventions for AP prevention.

Studies on compensatory strategies examined techniques such as the chin‐down posture^15^ and food texture modifications,^16^, ^17^ which effectively reduced aspiration risk. Research on compensatory postures for patients with ALS^23^ and oropharyngeal resistance therapy in older dysphagia patients^24^ also showed promising results. Exercise‐based interventions enhanced oral intake ability,^18^, ^19^, ^20^ but none of these studies demonstrated a clear preventive effect on AP.

Additionally, 6 studies explored novel therapeutic approaches, including transcranial magnetic stimulation,^25^, ^26^, ^27^ neuromuscular electrical stimulation,^28^ and expiratory muscle strength training.^29^, ^30^ While these interventions demonstrated improvements in swallowing function and respiratory parameters, none directly assessed their effectiveness in AP prevention.

To summarize the effects of swallowing rehabilitation on AP from previous studies, compensation strategies effectively reduce aspiration risk, whereas exercise training primarily improves swallowing function, but there is limited evidence to support their role in preventing AP.

Our findings can be contextualized within existing clinical guidelines for AP management. The BTS Clinical Statement on Aspiration Pneumonia^1^ provides detailed recommendations, emphasizing the limited direct evidence supporting swallowing rehabilitation for AP prevention, and instead recommends a comprehensive management approach that includes oral care, careful risk assessment, and multidisciplinary intervention. The BTS guidelines recognize the potential value of compensatory strategies while noting the insufficient evidence base for exercise‐based approaches in AP prevention.

The JRS Guidelines for the Management of Pneumonia in Adults^2^ also acknowledge swallowing rehabilitation in their recommendations, though with less specific guidance on implementation approaches. This reflects the general recognition of swallowing management as a component of care, even as the evidence base continues to develop.

Our review findings are consistent with these guideline approaches, particularly the cautious stance of the BTS, highlighting that while swallowing rehabilitation shows promise, the evidence base remains limited, particularly for exercise‐based interventions. The current evidence most strongly supports compensatory strategies, which align with clinical practice guidance. These findings underscore the need for more robust research to better inform future guideline recommendations in this important area of patient care.

A review by Teramoto^31^ from Japan highlighted the need for a comprehensive approach to oropharyngeal dysphagia management in AP patients, integrating swallowing rehabilitation and oral care. However, it lacked specific evidence supporting swallowing rehabilitation, reflecting a broader challenge—as revealed in our current review—while comprehensive dysphagia management is widely recommended, clear evidence for exercise‐based approaches in AP remains limited.

In addition to the swallowing rehabilitation interventions covered in our review, various preventive strategies for AP have been explored. A systematic review by Santos et al.^32^ examined interventions for preventing AP in older adults, identifying 13 studies, including 6 on pharmacological interventions, 3 on compensatory strategies, and others on oral care, screening methods, and multidisciplinary interventions. Positive outcomes in pneumonia prevention were demonstrated in three pharmacological intervention studies^33^, ^34^, ^35^ and one oral hygiene study.^36^ The BTS Clinical Statement cites evidence from studies such as Yoneyama et al.,^37^ emphasizing the role of oral care in pneumonia prevention. These findings, combined with our review results, suggest that AP prevention should be optimized not by overemphasizing swallowing rehabilitation (exercise training and compensatory strategies), which has insufficient evidence of effectiveness, but by integrating it with other preventive measures such as oral hygiene protocols and pharmacological interventions.

These observations suggest several priorities for future research. First, well‐designed prospective studies are needed to evaluate the effectiveness of exercise‐based approaches, specifically in patients with AP. While current studies indicate potential benefits for swallowing function and oral intake capacity, long‐term follow‐up studies should assess their impact on AP prevention. These studies must clearly describe intervention protocols and differentiate between compensatory and exercise‐based components. Second, research should define target populations more precisely and analyze outcomes separately for different patient groups, considering the diverse causes and presentations of AP.

The findings of this review suggest that clinical practice should prioritize established compensatory strategies and integrate exercise‐based techniques based on individual patient factors and underlying diseases. In light of the findings of Komiya et al.^3^ that the majority of older pneumonia patients in Japan are managed by non‐pulmonologists in daily clinical practice, this suggestion will be useful in creating practical swallowing rehabilitation protocols that can be effectively implemented in primary care settings, where many patients with AP first visit and receive ongoing care.

This review has some limitations. First, our literature search was restricted to MEDLINE and the Cochrane Library databases. We selected these major freely accessible databases to enhance the universality and reproducibility of our review, aiming to ensure that our search strategy could be readily replicated by researchers globally without subscription barriers. While this approach is consistent with scoping review methodology and ensured comprehensive coverage of high‐quality English‐language publications, we acknowledge that excluding EMBASE, CINAHL, Web of Science, and Japanese‐language databases might have resulted in the omission of relevant studies, particularly from the substantial body of Japanese research on dysphagia rehabilitation. Additionally, our search strategy might have caused the exclusion of relevant studies using different terminology for swallowing rehabilitation or AP. We also acknowledge that, in accordance with our inclusion criteria, systematic reviews and meta‐analyses were excluded from this scoping review. Therefore, some recent review‐level evidence and Japanese‐language research might not be reflected in our findings. Future reviews incorporating broader databases and languages are warranted to achieve a more comprehensive synthesis of the literature. Second, while supplementary studies on dysphagia rehabilitation in other populations were included, their analysis was not exhaustive. Third, the heterogeneity in outcome measures and intervention descriptions made quantitative synthesis challenging. Furthermore, while this review incorporated several key review articles and guidelines excluded in the initial screening process, additional relevant literature may exist that could provide further insights.

This review did not conduct a formal quality appraisal or risk of bias assessment of individual studies, in accordance with the Arksey and O'Malley framework for scoping reviews. As per this methodology, our primary aim was to map the extent and nature of available evidence rather than to evaluate intervention efficacy. While this approach is appropriate for the scoping nature of our review, it means that readers should interpret the strength of evidence with caution. As is standard in scoping reviews, we did not conduct a formal quality assessment of included studies. However, many of the included studies were retrospective and heterogeneous; therefore, the findings should be interpreted with particular caution. The lack of quality assessment limits our ability to make definitive conclusions about the effectiveness of various interventions. Future systematic reviews focusing on specific interventions identified in this review may benefit from incorporating critical appraisal to provide more robust evaluations of effectiveness.

## 5 CONCLUSION

This scoping review identified limited evidence for swallowing rehabilitation in patients with AP. While compensatory strategies are effective, current evidence does not support the use of exercise training for AP prevention. Future studies should use prospective studies, clearly define intervention protocols, and differentiate compensatory from exercise‐based strategies to assess their role in AP prevention.

In clinical practice, compensatory strategies should remain central due to the lack of evidence supporting the routine integration of exercise‐based approaches for AP prevention. These should be complemented by oral care and pharmacological interventions tailored to individual patient needs.

## AUTHOR CONTRIBUTIONS

**Akihito Ueda:** Conceptualization; investigation; formal analysis; data curation; writing – original draft. **Kanji Nohara:** Writing – review and editing; supervision; investigation.

## FUNDING INFORMATION

The authors received no specific funding for this work.

## CONFLICT OF INTEREST STATEMENT

The authors declare no conflict of interest.

## ETHICS STATEMENT

Ethics approval statement: None.

Patient consent statement: None.

Clinical trial registration: None.

## Supporting information


Data S1.

## Data Availability

The data supporting this study were obtained from publicly available databases (MEDLINE and Cochrane Library), as detailed in the Methods section. The complete search results and data extraction sheets are available from the corresponding author upon reasonable request.
